# Draft Genome Sequence of Atrazine-Utilizing Bacteria Isolated from Indian Agricultural Soil

**DOI:** 10.1128/genomeA.01149-13

**Published:** 2014-01-09

**Authors:** Sneha Sagarkar, Pooja Bhardwaj, Trilok C. Yadav, Asifa Qureshi, Anshuman Khardenavis, Hemant J. Purohit, Atya Kapley

**Affiliations:** Environmental Genomics Division, National Environmental Engineering Research Institute, CSIR-NEERI, Nagpur, India

## Abstract

We report the draft genome sequences of two tropical bacterial isolates capable of degrading the herbicide atrazine. *Alcaligenes* sp. strain EGD-AK7 and *Arthrobacter* sp. strain AK-YN10 were isolated from Indian agricultural soil in which sugarcane is grown, with a reported history of atrazine use. EGD-AK7 has the *atzABCDEF* genes and AK-YN10 has the *trzN* and *atzBC* genes for atrazine degradation.

## GENOME ANNOUNCEMENT

Atrazine (2-chloro-4-ethylamino-6-isopropylamino-1,3,5-triazine) is a member of the s-triazine group of herbicides. Due to their excessive usage, high persistence, and mobility, atrazine and its metabolites are found in soil and water bodies. The accumulation of atrazine in the environment is of great concern, as several studies have shown that atrazine is a potent endocrine disruptor (1).

This study reports the draft genomes of two bacteria that demonstrate the potential for atrazine bioremediation. These isolates follow diverse atrazine degradation pathways. The bacteria were grown in basal salt medium at 30°C and the total DNA was prepared as reported earlier (2). The genomes were sequenced using the Illumina MiSeq (Applied Biosystems) sequencing platform; the reads were assembled using GS Assembler version 2.6. The genome was annotated using the NCBI Prokaryotic Genomes Automatic Annotation Pipeline (PGAAP) and was independently analyzed on the RAST server (3). Although all genes from the atrazine degradation pathway were not annotated in the draft genome, they have been individually cloned from the respective isolates and their presences confirmed by sequencing.

The sequence of 16S rRNA gene (1,492 bp) of *Alcaligenes* sp. strain EGD-AK7 is 100% identical to that of *Alcaligenes* sp. strain HPC1271 (accession no. AMXV01000004) (4). The atrazine degradation genes present in this bacterium are *atzA*, *atzB*, *atzC*, *atzD*, *atzE*, and *atzF*, which are homologous to the *atz* genes from the ADP plasmid in *Pseudomonas citronellolis*. The 4.28-Mb draft genome of this bacterium was assembled into 70 contigs with 56.6% G+C content. Fifty-three tRNAs, 10 rRNAs, and 4,054 genes were annotated.

Annotation by RAST revealed that 80 genes were grouped under the metabolism of aromatic compounds subsystem and 406 genes were grouped as amino acid and derivatives, synthesis, and metabolism.

The sequence of the 16S rRNA gene (1,487 bp) of *Arthrobacter* sp. strain AK-YN10 demonstrates 98% similarity to that *Arthrobacter aurescens* TC1 (5). The 4.84-Mb draft genome was assembled into 107 contigs with 63.3% G+C content. Fifty-five RNAs and 4,634 genes were annotated. This bacterium contains the *trzN*, *atzB*, and *atzC* genes from the atrazine degradation pathway. The *atzB* and *atzC* genes are present on contigs 63 and 35, respectively. These genes are homologous to the *atzB* and *atzC* genes of TC1 plasmid of *A. aurescens* TC1.

Apart from atrazine-degrading genes, many metal resistance genes, antibiotic resistance genes, dioxygenases, monooxygenases, and mobile genetic elements were also annotated in the draft genome of isolate AK-YN10. Annotation by RAST revealed that 552 genes are grouped under carbohydrate metabolism.

### Nucleotide sequence accession numbers. 

The whole-genome shotgun sequences were deposited in GenBank under accession numbers AVOG00000000 (version AVOG02000000) for *Alcaligenes* sp. EGD-AK7 and AVPD00000000 (version AVPD01000000) for *Arthrobacter* sp. AK-YN10.
